# The *ACE* gene D/I polymorphism as a modulator of severity of cystic fibrosis

**DOI:** 10.1186/1471-2466-12-41

**Published:** 2012-08-08

**Authors:** Fernando A L Marson, Carmen S Bertuzzo, Taís D R Hortencio, José D Ribeiro, Luciana C Bonadia, Antônio F Ribeiro

**Affiliations:** 1 Department of Pediatrics, School of Medical Sciences, University of Campinas, P.O. Box: 6111, Campinas, SP, 13081-970, Brazil; 2 Department of Genetics, Faculty of Medical Sciences, University of Campinas, P.O. Box: 6111, Campinas, SP, 13081-970, Brazil

**Keywords:** Genotype, Phenotype, Variability, Genetic modulation, Angiotensin-converting Enzyme

## Abstract

**Background:**

Cystic Fibrosis (CF) is a monogenic disease with complex expression because of the action of genetic and environmental factors. We investigated whether the *ACE* gene D/I polymorphism is associated with severity of CF.

**Methods:**

A cross-sectional study was performed, from 2009 to 2011, at University of Campinas – UNICAMP. We analyzed 180 patients for the most frequent mutations in the *CFTR* gene, presence of the *ACE* gene D/I polymorphism and clinical characteristics of CF.

**Results:**

There was an association of the D/D genotype with early initiation of clinical manifestations (OR: 1.519, CI: 1.074 to 2.146), bacterium *Burkholderia cepacia* colonization (OR: 3.309, CI: 1.476 to 6.256) and Bhalla score (BS) (p = 0.015). The association was observed in subgroups of patients which were defined by their *CFTR* mutation genotype (all patients; subgroup I: no mutation detected; subgroup II: one *CFTR* allele identified to mutation class I, II or III; subgroup III: both *CFTR* alleles identified to mutation class I, II and/or III).

**Conclusion:**

An association between the D allele in the *ACE* gene and the severity of CF was found in our study.

## Background

*CFTR* gene mutations are crucial in modulating the severity of cystic fibrosis (CF), along with environmental factors and modifier genes [1-7]. CF occurs with heterogeneous clinical presentation. Among the clinical symptoms, that of highest variability is lung disease [5], and modifier genes have been analyzed and associated as possible factors that influence this clinical response [3,7].

The *ACE* gene codifies the Angiotensin Converting Enzyme (ACE). Based on the pro-inflammatory property of the ACE protein [8,9], the *ACE* gene was selected as a possible genetic marker for clinical denotation in CF. The ACE enzyme catalyzes the conversion of angiotensin I to angiotensin II peptide, acting in the blood pressure control and the electrolyte balance of blood, being an important vasoconstrictor and stimulant of aldosterone [8,10].

The *ACE* gene is located on region 17q23.3 [11]. A biallelic polymorphism, named as I (insertion) and D (deletion), with D allele characterized by a deletion of the 287 pb DNA fragment in intron 16, affects the level of the ACE enzyme. The polymorphism determines the amount of ACE enzyme in the plasma and tissues [8,10,12]. Individuals with I/I genotype have low concentrations of ACE; with D/D genotype, higher concentrations; and with D/I genotype, intermediate.

The aim of this study was to investigate the association of the *ACE* gene D/I polymorphism and *CFTR* genotype with the severity of CF, determined by twenty four clinical markers of the disease.

## Methods

We conducted a cross-sectional study with patients from the CF Specialized Center at the University of Campinas - UNICAMP, in a period from 2009 to 2011. Diagnosis of CF was confirmed in patients through two doses of sodium and chloride from the sweat with values greater than 60 mEq/L. In a cohort of patients we identified two mutations in the *CFTR* gene. No patient had received the neonatal screening test performed for CF.

Two hundred and fifteen patients were selected for the study. Thirty five patients without clinical data for statistical analysis and those who did not sign the consent form were excluded. Patients' DNA was obtained by phenol-chloroform extraction. The concentration of DNA used for analysis was 50 ng/mL, evaluated using GE NanoVue^TM^ Spectrophotometer (GE Healthcare Biosciences, United States of America, Pittsburgh).

### Determination of mutations in the *CFTR* gene

Determination of mutations in the *CFTR* gene was performed in the Laboratory of Molecular Genetics for mutations by polymerase chain reaction (F508del) and restriction fragment length polymorphism method (G542X, R1162X, R553X, G551D and N1303K). Some mutations in patients with CF were obtained by sequencing or MLPA (Multiplex Ligation-dependent Probe Amplification) analysis: S4X, 2183A > G, 1717-G > A and I618T. For sequencing and MLPA, we used the same MegaBace1000® property (GE Healthcare Biosciences, United States of America, Pittsburgh) [13]. The *CFTR* genotype was used as a correction factor for statistical analysis. All mutations identified were included in classes one, two or three of the *CFTR* gene. Others identified mutations as class IV (P205S e R334W) were included in the statistical analysis in the not identified mutation subgroup, to minimize the associated factor with the mutation classes in the *CFTR* gene, being that the class IV is associated with a minor severity.

### *ACE* gene D/I polymorphism analysis

ACE*D and ACE*I were identified by amplifying the respective fragments from intron 16 of the *ACE* gene. The PCR reaction contained 25μL with 100 ng of DNA, 1 μM of each primer, 200 μM of deoxynucleotide triphosphate, 1.3 mM of MgCl_2_, 50 mM of KCl, 10 mM of Tris–HCl (pH 8.4 at 25°C), 0.1% of Triton X-100 and 0.35U of Taq DNA polymerase. A pair of primers (hace3s, 5'-GCC CTG CAG GTG TCT GCA GCATGT-3'; hace3as, 5'-GGA TGG CTC TCC CCG CCT TG TCTC-3') was used to amplify ACE*D and ACE*I, resulting in 319 bp and 597 bp, respectively [8,10,12,14]. The procedure for thermal cycling consisted of initial denaturation at 94°C for 7 min, subsequent denaturation at 94°C for 30 min, annealing at 56°C for 45 min, and extension at 72°C for 2 min, repeated for 35 cycles followed by a final extension at 72°C for 7 min. After the addition of 5 μL of glycerol-based loading buffer, 7 μL of the reaction was applied in agarose gel containing 1.5% of acetate, 40 mM of TRIS, 2 mM of EDTA and 1 μg of ethidium bromide per milliliter [8,10,12,14].

Due to the preferential amplification of the ACE*D in heterozygous individuals, each initial sample with a D/D genotype was passed through a second PCR reaction. Primers used: Hace 5a, 5'-TGG GAC CAC-AGC GCC CGC CAC TAC-3' and hace 5c, 5'-TCG CCA GCC CTC CCA TGC CCA TAA-3'. The PCR conditions were identical, except for the annealing temperature of 67°C. A 335 bp sequence was amplified in the presence of at least one allele [8,10,12,14].

### Clinical markers of severity of disease

Clinical scores of Kanga, Shwachman-Kulczycki and Bhalla (BS) were performed blindly by three qualified professionals from UNICAMP. These scores measure the pulmonary exacerbation, severity of CF and structural impairment of the lung, respectively [15]. Nutritional status was obtained by calculating the Body Mass Index (BMI) for age using the programs WHO Anthro [16], for patients up to five years old, and WHO Anthro Plus [17], for patients aged 5 to 19 years old. For patients older than 19 years old, the BMI was calculated [18]. Age of the diagnosis, the onset of pulmonary and digestive symptoms, and the first isolation of *Pseudomonas aeruginosa*, were used as markers of initiation of the disease. Results of cultures of sputum, performed during routine diagnosis for the mucoid and non-mucoid bacteria *P. aeruginosaStaphylococcus aureusBurkolderia cepacia* (BC) and *Achromobacter xylosoxidans* were included. Spirometry was performed in the Laboratory of Pulmonary Physiology (LAFIP) according the standards of the American Thoracic Society [19]. Parameters analyzed were: forced expiratory volume in 1 second (FEV_1_), forced vital capacity (FVC), FEV_1_/FVC ratio, forced expiratory flow between 25-75% and transcutaneous oxygen saturation (SaO2). For analysis of the spirometry data, we used the predicted value in%. Comorbidities (nasal polyposis, osteoporosis, diabetes mellitus, pancreatic insufficiency and meconium ileus) were also analyzed.

### Statistical analysis

Variables described for the onset of illness (age at diagnosis, onset of pulmonary and digestive symptoms and first isolation of *P. aeruginosa*) were categorized into two groups according to the median of the data, due to a non-normal distribution of data. Data categorized by the median are divided into two cohorts with similar sample size. For clinical evaluation of the scores from the SaO2 and spirometry tests, analyses were performed without adjusting the data. Bacteria isolated from the culture of airway secretions were used as markers according to the presence or absence of bacteria in three consecutive cultures in the past two years. Comorbidities were compared in terms of presence or absence. Statistical analyses was performed using the Statistical Package for the Social Sciences (SPSS) v.17.0 [20] and the R program version 2.12 (Comprehensive R Archive Network, 2011). In order to avoid spurious data due to the problem of multiple testing [21], the level of significance α, was adjusted using the Bonferroni correction for four tests. Calculation of statistical power for the sample, carried out by software GPOWER 3.0.5 [22], showed a statistical power above 80% for the analysis performed.

Data were compared by the linear and logistic regression analysis. For comparison between genotypes and the variables with numerical distribution, T-student test was applied to normal data distribution or Mann–Whitney test to non-normal data distribution. Genotyped data for the *CFTR* gene was used to establish an association between the *CFTR* gene, *ACE* gene and clinical variables. All mutations analyzed in our study were included in classes I, II or III. In the analyzed sample, four different analyses were performed in order to detail the effect of the genotype of the *CFTR* gene in clinical severity. The analyses were performed in the cohorts: (1) all patients with CF (180 patients); (2) patients with no identified mutation in the *CFTR* gene (44 patients); (3) patients with a mutant allele identified in the *CFTR* gene belonging to class I, II or III mutation(51 patients); and (4) patients with two mutations identified in the *CFTR* gene belonging to class I, II and/or III (85 patients) - main cohort to analyze the influence of modifier genes associated with clinical variation in CF.

This study was approved by the Institutional Ethics Committee from University of Campinas (Faculty of Medical (No. 528/2008), and all patients signed a consent form before beginning the study.

## Results and discussion

From the sample of 180 analyzed patients, 90 (50%) were male, 165 (91.7%) were European-Caucasian derived and 15 (8.3%) were African-derived individuals. The patients’ *CFTR* genotypes were: 44 patients (24.44%) without identified mutation, 51 (28.33%) with one identified mutation (25% F508del/-, 2.78% G542X/-, 0.56% R1162X/-) and 85 (47.22%) patients with two identified mutations (31.67% F508del/F508del, 6.67% F508del/G542X, 2.78% F508del/R1162X, 2.22% F508del/N1303K, 0.56% F508del/R553X, 0.56% F508del/S4X, 0.56% F508del/1717-1 G > A, 0.56% G542X/R1162X, 0.56% G542X/I618T, 0.56% G542X/2183A > G and 0.56% R1162X/R1162X).

The spectrum of isolated Bacteria in secretion was: 76 (42.2%) with mucoid and 101 (56.1%) with non-mucoid *P. aeruginosa*; 141 (78.3%), *S. aureus;* 25 (13.9%), *B. cepacia;* and 18 (10%), *A. xylosoxidans*. Comorbidities associated with CF severity were: 143 (79.4%) with pancreatic insufficiency; 33 (18.3%), nasal polyps; 33 (18.3%), diabetes mellitus; 29 (16.1%), osteoporosis; and 27 (15%), meconium ileus. For the variables with their numerical distribution, see data listed in Table 1.

**Table 1:** Description of quantitative variables (in months) of CF patients treated at the pediatric clinic at UNICAMP

**Variable**	N*	**Minimum**	**Maximum**	**Median**	**Mean**	**Standard error**	**Standard deviation**
Age	179	7	288	154	212.64	14.13	189.04
Onset of the manifestation	170	0	156	3	34.69	8.33	108.54
Age at diagnosis	173	0	170	24	91.47	12.44	163.60
Onset of digestive symptons	150	0	150	3	40.69	8.93	109.32
Onset of lungs symptons	165	0	156	6	42.88	9.24	118.68
1^st^. *P. aeruginosa*	131	6	180	31	102.60	15.16	173.47

*N- number of patients.

The *ACE* gene D/I polymorphism showed a higher frequency for ACE*D (228/360 alleles) compared with ACE*I (132/360 alleles). The genotype frequencies were: 72 (40.0%) with D/D; 84 (46.67%) with D/I; and 24 (13.3%) with I/I. The population is in Hardy-Weinberg equilibrium (p > 0.05). Analysis of 70 healthy control subjects in UNICAMP demonstrated the genotype frequency: 20 (29%) with D/D, 37 (53%) of D/I, and 13 (18%) I/I [23]. There was no difference in frequency of genotypes in relation to our study (p = 0.210). The analyses of the *ACE* gene D/I polymorphism with the clinical variables are denoted in Table 2, where every association possible between the clinical trial, *CFTR* mutation identified and *ACE* gene D/I polymorphism can be observed.

**Table 2:** **Association of*ACE*gene D/I polymorphism with variables used as markers of severity of CF, patients followed at the pediatric center in UNICAMP distributed by*CFTR*gene mutation identified divided into cohorts**

**Variable**		**Without taking* CFTR *mutation into account**		**No identified mutation**		**One identified mutation**		**Two identified mutation**	
		**E**	**p**	**E**	**p**	**E**	**p**	**E**	**p**
Patients age		W:0.791	0.374	W:3x10^-5^	0.995	W:2.969	0.085	W:0.001	0.984
Onset of clinical manifestations		W:0.116	0.733	W:0.162	0.687	**W:4.29**	**0.038**	W:0.937	0.333
Diagnostic		W:0.11	0.74	W:0.047	0.83	W:0.099	0.753	W:0.087	0.768
Onset of digestive symptons		W:1.494	0.221	W:0.148	0.7	W:0.297	0.586	W:0.979	0.322
Onset of lung symptons		W:0.021	0.885	W:0.039	0.843	W:0.401	0.526	W:1.302	0.31
BMI		W:1.169	0.28	W:0.687	0.407	W:0.436	0.509	W:2.498	0.114
Nasal poliposys		W:0.62	0.431	W:0.984	0.321	W:0.419	0.517	W:1.26	0.262
Diabetes		W:0.358	0.55	W:0.016	0.901	W:0.174	0.676	W:0.184	0.668
Osteoporosis		W:0.877	0.349	W:1.056	0.0304	W:0.561	0.454	W:0.083	0.773
Pancreatic insuficience		W:1.6	0.206	W:0.693	0.406	W:1.063	0.302	W:0.182	0.669
Meconium ileus		W-0.252	0.616	W:3.813	0.051	W:1.109	0.292	W:1.498	0.221
SaO2		F:2.131	0.142	F:0.022	0.884	F:1.868	0.178	F:1.344	0.25
Scores	Bhalla	**F:6.526**	**0.012**	F:0.2	0.689	**F:4.942**	**0.032**	**F:4.013**	**0.049**
	Kanga	F:1.3	0.256	F:0.486	0.492	F:0.027	0.871	F:3.765	0.057
	SK	F:2.361	0.127	F:0.286	0.597	F:1.042	0.312	F:1.243	0.269
FVC		F:0.139	0.71	F:0.829	0.37	F:0.918	0.345	F:0.93	0.339
FEV_1_		F:0.785	0.377	F:0.622	0.436	F:0.907	0.348	F:2.797	0.099
FEV_1_/FVC		F:0.891	0.347	F:0.005	0.943	F:2.212	0.146	F:0.156	0.694
FEF25-75%		F:0.42	0.518	F:0.112	0.735	F:0.02	0.887	F:1.048	0.31
1^a^*P. aeruginosa* isolated		W:0.962	0.327	W:0.702	0.402	W:0.16	0.69	W:0.099	0.753
Isolated Bacteria	PAM	W:0.92	0.338	W:0.141	0.708	W:2.016	0.156	W:0.165	0.684
	PANM	W:1.21	0.272	W:1.149	0.284	W:0.987	0.753	W:0.262	0.609
	AX	W:3.2	0.074	W:0.038	0.845	W:0.642	0.423	W:2.911	0.088
	BC	**W:4.290**	**0.038**	W:0.1	0.753	W:3.681	0.055	W:0.341	0.559
	SA	W:0.209	0.65	W:1.151	0.283	W:0.191	0.662	W:1.102	0.294

Analysis by linear regression (F) and logistic regression (W) test . Values below of 0.05 for p denote a clinical correlation between variables. E - Statistical,% - percentage, SaO2 - transcutaneous oxygen saturation, FEV_1_ – forced expiratory volume in 1 second, FVC – forced expiratory capacity, FEV_1_/FVC – ratio between two variables, forced FEF25-75, SC - expiratory flow at 25-75% of the pulmonary volume, SK - Shwachman-Kulczycki, PAM – *P. aeruginosa* mucoid, PAM – *P. aeruginosa* non mucoid, AX – *A. xylosoxidans*, BC – *B. cepacia*, SA – *S. aureus, BMI –* body mass index, CF - cystic fibrosis, ACE – angiotensin converting enzyme. No identified mutation (44 patients) – patients without of identified mutation in classes I, II or III. One identified mutation (51 patients) – patient with one identified mutation in class I, II, or III. Two identified mutation (85 patients) – patient with two mutations in class I, II and/or III. Others identified mutations as class IV (P205S e R334W) was included in the statistical analysis in the not identified mutation subgroup, to minimize the associated factor with the mutation classes in the *CFTR* gene.

The *ACE* gene D/I polymorphism was associated with the onset of clinical manifestations (Table 3), in the subgroup of patients with one identified *CFTR* mutation. We observed that patients with I/I genotype had OR: 0.297 (0.084 – 0.995), as protection factor, and the ones with D/D genotype had OR: 1.519 (1.074 to 2.146), as a severity factor.

**Table 3:** **Association of*ACE*gene D/I polymorphism with onset of clinical symptoms of patients in months considering the cohorts to*CFTR*mutation**

**Groups**	***ACE*genotype**	**≤ 3 months**	**> 3 months**	**Total**	**X**^**2**^	**p**	**X**^**2**^	**p**	**OR (CI 5-95%)**
Without taking *CFTR* mutation into account	I/I	38	28	66	0,880	0,644	0.012	0.9136	1.035 (0.555 - 1.931)
	I/D	44	37	81			0.473	0.492	0.808 (0.4396 -1.484)
	D/D	15	8	23			0.723	0.395	1.486 (0.5937, 3.721)
No identified *CFTR* mutation	I/I	13	6	19	1,685	0,431	0.825	0.364	1.806 (0.5017 -6.498)
	I/D	9	9	18			1.624	0.203	0.438 (0.122 - 1.576)
	D/D	3	1	4			0.357	0.977	2.045 (0.194 - 21.58)
One *CFTR* mutation identified class I, II or III	I/I	10	11	21	**5,564**	**0,062**	**4.217**	**0.049**	**0.297 (0.084 – 0.995)**
	I/D	14	6	20			0.521	0.471	1.167 (0.775 – 1.757)
	D/D	8	1	9			2.951	0.097	5,667 (0,647 – 49,61)
Two *CFTR* mutation identified class I, II and/or III	I/I	15	11	26	0,599	1,026	0.773	0.379	1.527 (0.593 - 3.936)
	I/D	21	22	43			0.122	0.727	0.854 (0.352 - 2.072)
	D/D	4	6	10			0.511	0.704	0.611 (0.158 - 2.358)

Values below 0.05 for p denote clinical correlation between variables. OR - odds ratio, CI- confidence interval, ≤ − less than or equal to, > − greater, D - deleted allele, I - insertion allele. No identified mutation (44 patients) – patients without of identified mutation in classes I, II or III. One identified mutation (51 patients) – patient with one identified mutation in class I, II, or III. Two identified mutation (85 patients) – patient with two mutations in class I, II or III.

The ACE*D is associated with a higher gene expression and, consequently, promotes a greater inflammatory response in the body, leading to early symptoms [8,10,12,14,24]. The earliest onset of signs and symptoms are accompanied by early onset of inflammation and deterioration of lung and pancreatic functions. These symptoms are characteristic of severe patients.

An association of the infection/colonization by *B. cepacia* with *ACE* gene D/I polymorphism was identified for patients without taking the *CFTR* mutation into account, OR: 4.509 (1.513 - 10.89), and for patients with one *CFTR* mutation identified to class I, II or III, OR: (1.43 - 40.38), for the D/D genotype (Table 4).

**Table 4:** **Association of the*ACE*gene D/I polymorphism, without*CFTR*genotype distribution and presence of*B. cepacia*(BC)**

**Group**	**Ace genotype**	**Presence**	**Absence**	**Total**	**X**^**2**^	**p**	**X**^**2**^	**p**	**OR (CI 5-95%)**
Without taking *CFTR* mutation into account	I/I	8	64	72	**8.654**	**0.013**	0.468	0.498	0.699 (0.319 – 1.534)
	I/D	9	74	82			0.814	0.182	0.651 (0.304 – 1.394)
	D/D	8	16	24			**8,653**	**0.003**	**4.509 (1.513 – 10.89)**
No identified *CFTR* mutation	I/I	3	18	21	0.530	0.767	0.003	1.29	1.056 (0.188 - 5.925)
	I/D	2	16	18			0.204	1.00	0.656 (0.107 - 4.041)
	D/D	1	3	4			0.438	0.93	2.267 (0.196 - 26.27)
One *CFTR* mutation identified class I, II or III	I/I	2	20	22	5.539	0.063	1.248	0.466	0.383 (0.069 - 2.117)
	I/D	2	18	20			0.787	0.629	0.463 (0.084 - 2.562)
	D/D	4	5	9			**6.834**	**0.027**	**7.6 (1.43 - 40.38)**
Two *CFTR* mutation identified class I, II or III	I/I	3	26	29	0.511	0.774	0.084	1.07	0.8077 (0.193 - 3.387)
	I/D	5	40	45			0.039	1.10	0.875 (0.234 - 3.275)
	D/D	2	9	11			0.495	0.764	1.833 (0.335 - 10.02)

Values below 0.05 for p denote clinical correlation between variables. OR - odds ratio, CI- confidence interval, D - deleted allele, I - insertion allele. No identified mutation (44 patients) – patients without of identified mutation in classes I, II or III. One identified mutation (51 patients) – patient with one identified mutation in class I, II, or III. Two identified mutation (85 patients) – patient with two mutations in class I, II or III.

**Figure 1:**
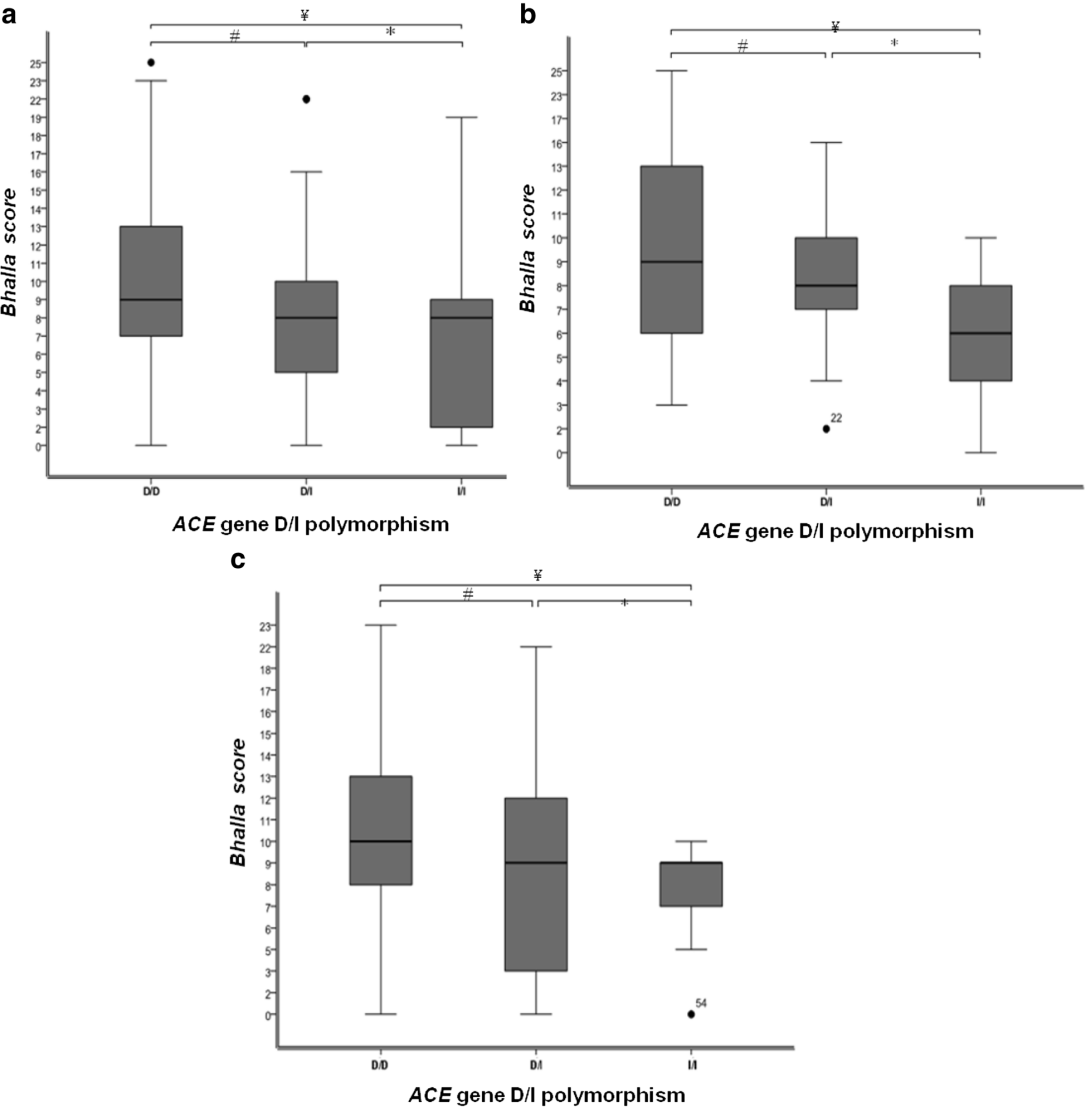
** Association of clinical data, with numerical distribution, with*ACE*gene D/I polymorphism and subgroups of*CFTR*mutations.** a Bloxplot denoting the association of the *ACE* gene D/I polymorphism in patients without taking *CFTR* gene into account. There were significant differences between groups of patients with the genotypes D/D and I/I. # p: 0.092 * p: 0.768, ¥ p: 0.045. **b.** Bloxplot denoting the association of the *ACE* gene D/I polymorphism in the subgroup of patients for whom one class I, II and III mutation have been identified. There were significant differences between groups of patients with genotypes D/D and I/I. # p: 0.478, * p: 0.183; ¥ p: 0.043. **c.** Bloxplot denoting the association of the *ACE* gene D/I polymorphism in the subgroup of patients for whom two class I, II and/or III mutation have been identified. There were significant differences between groups of patients with genotype D/D and I/I. # p: 0.789 * p: 0.505; ¥ p: 0.05. Analysis was performed by Mann–Whitney test considering a p-value of 0.05 as statistically significant

In the analysis of the BS and *ACE* gene D/I polymorphism, an association was found when no grouping by *CFTR* genotype occurred (p = 0.015), as well in the subgroup of patients for whom one class I, II and/or III mutation have been identified (p = 0.038), and in the subgroup of patients for whom two class I, II and/or III mutation have been identified (p = 0.042) (Figure 1). There was no difference between BS and the age of patients after categorization. Younger patients (≤ 154 months) had the same distribution of BS as older patients (> 154 months) (p = 0.761). Age is not a variable that contributes to the association between the *ACE* gene D/I polymorphism and BS. The analysis of an association between the BS and the age of patients with CF was performed in order to show that age had no influence on the score value analysis. We can conclude that the *ACE* gene D/I polymorphism acts in genetic modulation by association with BS. The BS is a computed tomography score, which measures pulmonary involvement, therapeutic effects and selection of patients for transplantation, which detects anatomical changes of the lung parenchyma [15,25]. The BS has low variation between examiners, good reproducibility, high sensitivity and specificity, and high correlation with pulmonary function test [15]. The values obtained in the score can predict severity associated with deterioration of the structure of the lung parenchyma, which later in clinical evolution can be observed by other variables such as BMI and lung function.

Evolution of CF is secondary to mutation class in the *CFTR* gene and environment factors. Many studies have correlated mutations, polymorphisms and clinical variables to CF [5,26]. Association studies commonly face the problem of having insufficient sample size for the number of mutations in the *CFTR* gene to achieve a homogeneous population and characterize the follow-up of chronic and persistent lung disease [27].

Unlike other genetic diseases such as asthma, CF is monogenic. It was expected that mutations in the *CFTR* gene would determine the CF severity. Patients with mutations of classes I, II and III have more severe clinical forms than those with mutations IV, V and VI. However, we can observe changes in severity of CF in patients with identical mutations in the *CFTR* gene [28]. Our study allowed us to characterize the association between the *CFTR* gene, the environment and one possible CF modifier gene in patients of a Reference University Center, using a statistical method of gene association versus clinical markers.

The main environmental factor in the clinical variability of CF is the patients ´ access to treatment [28]. At our center, treatment is guaranteed by the public health system, which allows equal access for all patients included in the study, and it is not an additional factor in the analysis of data, which is not true in all CF centers in Brazil. Unlike the U.S. where the private system ensures better treatment in CF [29], in Brazil, the public health system is the reference.

Some review articles have suggested a possible modulation of the CF severity by the *ACE* gene. This fact is based on the proinflammatory property of the ACE protein [2,3,30]. To the best of our knowledge, few studies had characterized the *ACE* gene as a potential factor in the clinical CF severity [8,30,31]. Bartlett *et al*. (2009) [31], in a multicenter study, studied the same polymorphism in relation to the propensity for liver disease in patients with CF. They genotyped 124 patients with CF and liver disease and 843 patients with CF and no liver disease. In addition to this polymorphism, four other genes, and their polymorphisms, were analyzed. The polymorphism D/I in the *ACE* gene was not associated with the presence of liver disease in CF patients, OR: 1.11 (0.85 to 1.44).

Finally, the presence of *B. cepacia* complex increases inflammation, favoring the exacerbation of immune response, further deterioration of the bronchopulmonary structure and causing rapid deterioration of lung function [32]. More studies to determine whether the presence of the D/D genotype causes increased gene expression and, therefore, facilitates chronic infection by different bacteria are needed. The D/D genotype of the *ACE* gene D/I polymorphism was significantly associated with higher values of BS. Higher values on the BS are associated with greater clinical severity [15]. Patients with the D/D genotype had higher severity, when compared to patients with the I/I genotype. This data confirms that higher gene expression, given by the D allele, leads to a change in the structure of the lung parenchyma, with subsequent increases in the value of the score.

Our data suggest that *ACE* gene should be studied in other populations, principally in populations with high prevalence of chronic pulmonary infection by *B. cepacia*, early onset of clinical manifestations and early onset of severe lung disease (showed by BS).

Patient’s subgroups that were defined on the basis of *CFTR* mutation analysis may also to be different in comorbidities which may unmask the role of the *ACE* gene as modifier that was studied in this study, being a limitation of our work.

## Conclusion

CF patients with the D/D genotype for the *ACE* gene D/I polymorphism have a higher risk for chronic infection with BC and deterioration of lung function, characterized by a high BS. There was an association between the presence of the D allele *ACE* gene and the severity of CF. Further studies are needed to verify the pro-inflammatory activity of this gene in CF, along with a larger CF population with homogeneous *CFTR* mutation; suggesting that in this case, a multicenter study is necessary.

## Competing interests

The authors declare that they have no competing interests.

## Authors’ contributions

FALM: made substantial contributions to conception and design, acquisition of data, and analysis and interpretation of data; involved in drafting the manuscript and revising it for critically important intellectual content. TDRH: participated in the design of the study and in the collection of clinical markers. CSB: carried out the molecular genetic studies and drafted the manuscript. AFR: has been involved in drafting the manuscript and revising it critically for important intellectual content. LCB: performed genotyping for *CFTR* mutation. JDR: has given final approval for the publishing of this version. All authors read and approved the final manuscript.

## Pre-publication history

The pre-publication history for this paper can be accessed here:

http://www.biomedcentral.com/1471-2466/12/41/prepub
